# Structural organization and sequence diversity of the complete nucleotide sequence encoding the *Plasmodium malariae* merozoite surface protein-1

**DOI:** 10.1038/s41598-022-19049-z

**Published:** 2022-09-16

**Authors:** Chaturong Putaporntip, Napaporn Kuamsab, Rattanaporn Rojrung, Sunee Seethamchai, Somchai Jongwutiwes

**Affiliations:** 1 Molecular Biology of Malaria and Opportunistic Parasites Research Unit, Department of Parasitology, Faculty of Medicine, Chulalongkorn University, Bangkok, Thailand; 2 Cannabis Health Sciences, College of Allied Health Sciences, Suan Sunandha Rajabhat University, Samut Songkhram, Thailand; 3 Department of Biology, Faculty of Science, Naresuan University, Pitsanulok, Thailand

**Keywords:** Microbiology, Molecular biology, Diseases

## Abstract

The merozoite surface protein-1 (MSP1) is a prime candidate for an asexual blood stage vaccine against malaria. However, polymorphism in this antigen could compromise the vaccine’s efficacy. Although the extent of sequence variation in *MSP1* has been analyzed from various *Plasmodium* species, little is known about structural organization and diversity of this locus in *Plasmodium malariae* (*PmMSP1*). Herein, we have shown that *PmMSP1* contained five conserved and four variable blocks based on analysis of the complete coding sequences. Variable blocks were characterized by short insertion and deletion variants (block II), polymorphic nonrepeat sequences (block IV), complex repeat structure with size variation (block VI) and degenerate octapeptide repeats (block VIII). Like other malarial *MSP1*s, evidences of intragenic recombination have been found in *PmMSP1*. The rate of nonsynonymous nucleotide substitutions significantly exceeded that of synonymous nucleotide substitutions in block IV, suggesting positive selection in this region. Codon-based analysis of deviation from neutrality has identified a codon under purifying selection located in close proximity to the homologous region of the 38 kDa/42 kDa cleavage site of *P. falciparum MSP1*. A number of predicted linear B-cell epitopes were identified across both conserved and variable blocks of the protein. However, polymorphism in repeat-containing blocks resulted in alteration of the predicted linear B-cell epitope scores across variants. Although a number of predicted HLA-class II-binding peptides were identified in PmMSP1, all variants of block IV seemed not to be recognized by common HLA-class II alleles among Thai population, suggesting that diversity in this positive selection region could probably affect host immune recognition. The data on structural diversity in *PmMSP1* could be useful for further studies such as vaccine development and strain characterization of this neglected malaria parasite.

## Introduction

Despite annual declines in global malaria cases caused by the two major human malaria parasites *Plasmodium falciparum* and *P. vivax* during the past 2 decades due to integrative control measures, an increase in the number of infections by the low prevalent species including *P. malariae* and *P. ovale* spp. has been observed in some African endemic areas, such as Tanzania, Gabon, Democratic Republic of Congo and Uganda^1–4^. Although *P. malariae* infection usually does not result in acute severe symptoms, repeated and long-term exposures may be associated with chronic glomerulonephritis in children and adolescents in some endemic areas, especially Sub-Saharan Africa and Papua New Guinea^5–8^. While more compelling evidences are required to document chloroquine-resistance in *P. malariae*, the blood stage infection of this *Plasmodium* species may persist for an unusually long period and can recrudesce after many years of dormancy^9–11^. Like other human malaria parasites, *P. malariae* has been incriminated in transfusion-transmitted malaria in which the prevalence seems to vary across endemic areas^12,13^. Meanwhile, the low parasite density of *P. malariae* among infected individuals has hampered efficient detection by conventional microscopy, especially when it co-infects with other malaria species^14–16^. On the basis of microscopy diagnosis, *P. malariae* infection accounted for approximately 0.1% of all malaria cases in Thailand^17^ whereas PCR could diagnose about five times higher than microscopic examination^15,18–20^. To achieve malaria control and elimination, it may require effective interventions against the low prevalent *Plasmodium* species including *P. malariae*.

One of the leading vaccine candidates against asexual blood stages of malaria parasites is merozoite surface protein-1 (MSP1) which is believed to play a crucial role in invasion of host erythrocytes by the merozoites and during their egression from infected cells after asexual reproductive maturation^21,22^. The MSP1 of *P. falciparum* (PfMSP1) is synthesized as a precursor protein during schizogony and subsequently processed into 4 polypeptides of 83, 30, 38 and 42 kDa. Prior to erythrocyte entry of the merozoites, secondary processing of the C-terminal 42-kDa fragment ensues, yielding 33- and 19-kDa protein fragments^23^. On the basis of amino acid sequence identity, PfMSP1 have been divided in to 17 blocks, containing five conserved, five semi-conserved and seven variable blocks^24^. The 19-kDa fragment containing two epidermal growth factor (EGF)-like domains has been considered to be a potential vaccine candidate because it is a target for invasion inhibitory antibodies while naturally acquired antibodies against this fragment have been associated with protection against symptomatic malaria among individuals living in malaria endemic areas^25,26^. Likewise, the tripeptide repeats in PfMSP1 could elicit protective antibodies among children in Sub-Saharan Africa^27^. Furthermore, erythrocyte-binding domains have been identified in the 83-, 38- and 33-kDa fragments of PfMSP1^28–31^. Meanwhile, the *MSP1* gene of *P. vivax* (*PvMSP1*) displays mosaic organization of variable blocks whereas those of *P. ovale* spp. (*PoMSP1*) and *P. knowlesi* (*PkMSP1*) exhibit structural variation that are different from that of *PfMSP1*^32–34^.

To date, mainly partial sequences of the *MSP1* gene of *P. malariae* (*PmMSP1*) have been determined using isolates from French Guiana, Cameroon, Brazil and Thai-Myanmar border which reveals conserved and variable blocks^35–39^. However, the organization of these blocks based on the complete coding sequences remains to be elucidated. Herein, we analyzed the complete coding sequence of *PmMSP1* among clinical isolates from diverse endemic areas of Thailand. Results have shown that *PmMSP1* contained four variable blocks flanked by five conserved blocks. Like other human malarial *MSP1*s, intragenic recombination and natural selection have influenced diversity at this locus^24,32–34^. Furthermore, analysis of predicted linear B-cell and helper T-cell epitopes has suggested that polymorphism in this protein could affect host immune recognition.

## Results

### Amplification and sequencing of *PmMSP1*

**Figure 1:**
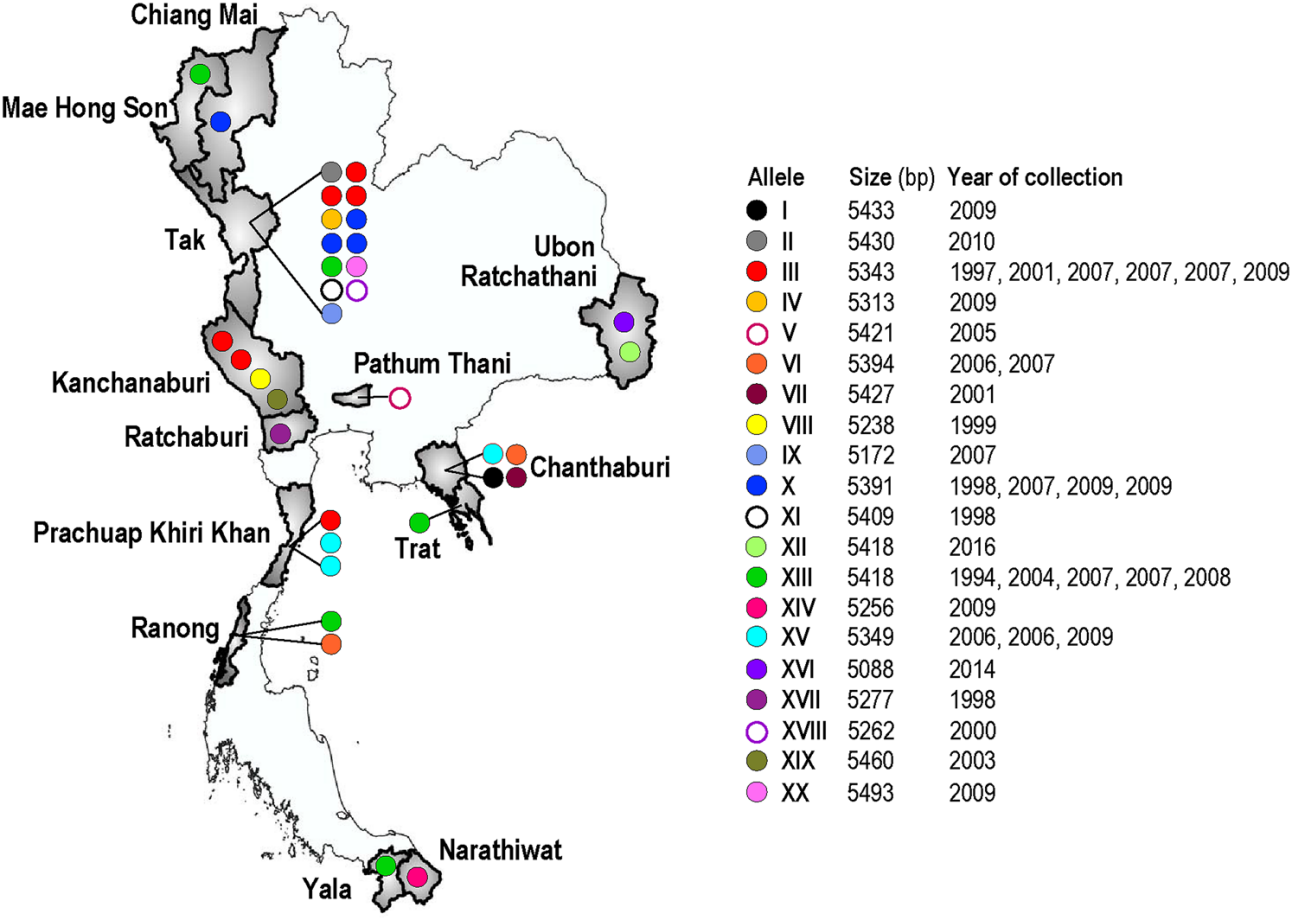
Map of Thailand showing the provinces and period of sample collection. Distribution of the *PmMSP1* alleles is shown as corresponding color circles. The map is modified from GADM maps and data (https://gadm.org/index.html) under the GADM license version 6.0.

The complete coding region of *PmMSP1* was amplified from 35 Thai isolates (PM1–PM35). The origins and years of sample collections are shown in Fig. 1. Of these, 15 isolates contained single infections of *P. malariae* and the remaining samples were co-infected with *P. falciparum* (n = 2), *P. vivax* (n = 17) and both *P. falciparum* and *P. vivax* (n = 1). However, the PCR primers used in this study were specific for amplification of *PmMSP1* because direct sequencing of the PCR-amplified products yielded clear electropherogram without superimposed signals of the sequences of this locus. Therefore, no cross amplification of *PfMSP1* and *PvMSP1* was observed in isolates containing *P. falciparum* or *P. vivax*. The complete coding sequences of *PmMSP1* in this study varied from 5088 to 5493 bp. In total, 20 alleles were identified in which alleles III, VI, X, XIII and XV contained more than one isolate (Fig. 1). Interestingly, the same alleles could be found from different sampling periods and from diverse endemic areas of the country. For example, allele XIII consisting of 5418 bp, occurred in five isolates from Mae Hong Son, Tak, Trat, Ranong and Yala Provinces collected during 1994, 2004, 2007 and 2008 (Fig. 1).

### Structural organization of *PmMSP1*

**Figure 2:**
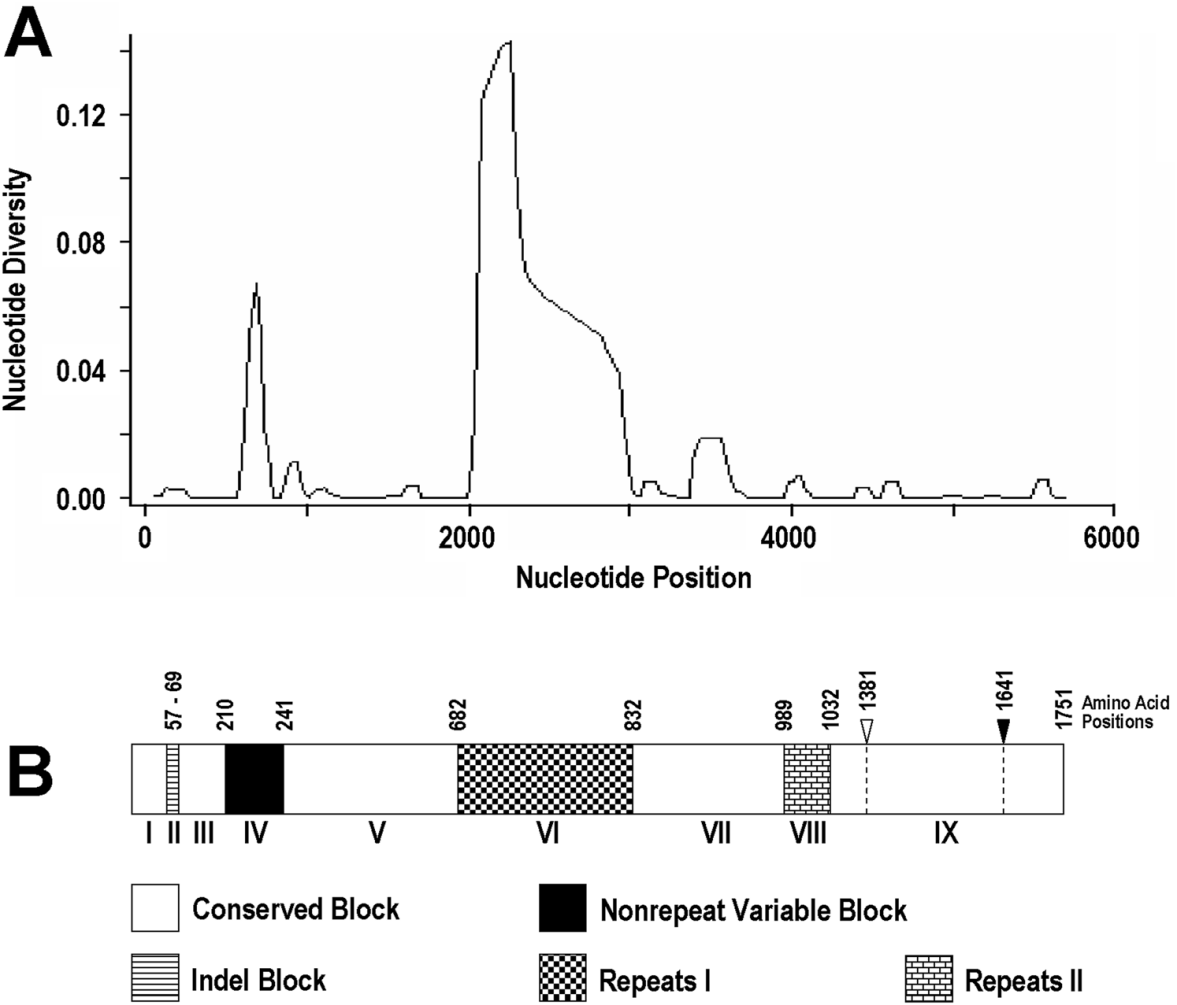
(**A**) Nucleotide diversity across the complete coding region of *PmMSP1* using window length of 100 nucleotides and step size of 25 nucleotides. (**B**) Corresponding scheme of PmMSP1 showing organization of the protein regions. The potential cleavage sites for the 42-kDa and 19-kDa fragments are shown in open and filled downward arrow heads, respectively.

**Figure 3:**
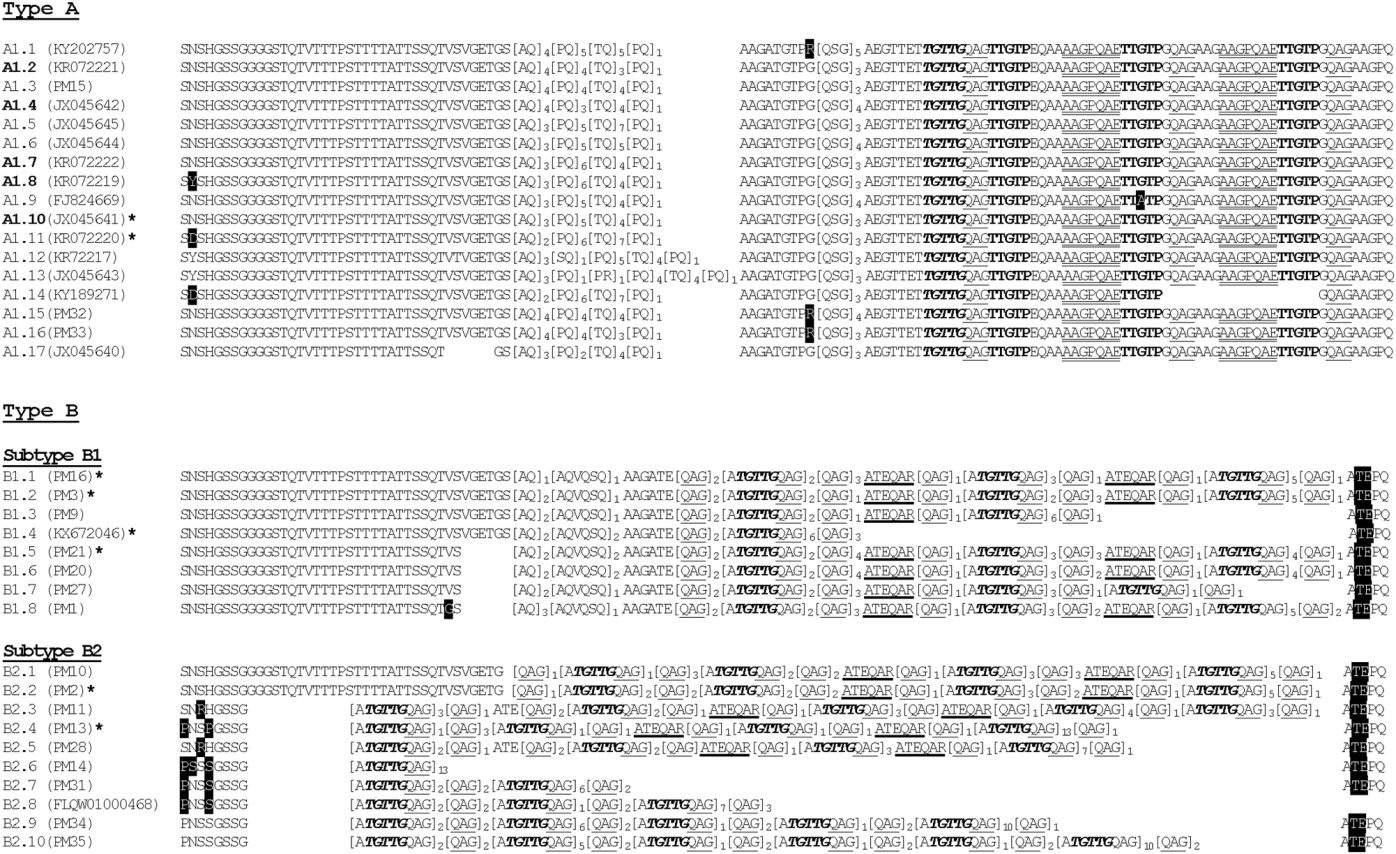
Variation in block VI of PmMSP1 spanning amino acid residues 683 and 827 (positions after GenBank accession no. FJ824669). Repeats are shown in brackets, bolds, italics or underlined residues. Numbers of repeat units are indicated by subscripts. Mutations in nonrepeat regions are highlighted. Representative isolates are shown in parentheses after the alleles. Alleles in bolds are found in *P. brasiliamum.* Allele A1.10 belongs to both *P. malariae* (KR072216) and *P. brasilianum* (JX045641). Asterisks denote alleles with two or more isolates: KR072218 and KR072215 in allele A1.11; PM17-PM19 in allele B1.1; PM3-PM7 in allele B1.2; KX672047 and KX672048 in allele B1.4; PM22-PM26 in allele B1.5; PM12 in allele B2.2; and PM29 and PM30 in allele B2.4.

To determine the structural organization of *PmMSP1*, nucleotide diversity was determined across the aligned complete coding sequences of 35 Thai isolates and the sequence from a Cameroonian patient (GenBank accession no. FJ824669) whose nucleotide and amino acid positions of the gene/protein were used as reference. Results revealed that the extent of nucleotide diversity was variable across *PmMSP1* with two regions containing nucleotide diversity > 0.04 (Fig. 2A), a comparable level for variable blocks of *PvMSP1*^32^. One was from codons 210 to 241, designated block IV, and the other was in block VI spanning amino acids 682 and 832. Short insertions and deletions (indels) were found between codons 57 and 69 at the N-terminal part of *PmMSP1*, designated block II, (Fig. 3). Meanwhile, Tandem Repeats Finder algorithm has identified two repeat-containing regions in *PmMSP1*, one corresponding to block VI and the other from codons 989 to 1032 (block VIII). Therefore, the remaining nonrepeat regions encompassing approximately 86% of the entire coding region in *PmMSP1* with nucleotide diversity < 0.02 were assigned to conserved blocks, consisting of blocks I, III, V, VII and IX (Fig. 2B).

### Diversity of indels in *PmMSP1*

Previous reports have shown that *P. malariae* and *P. brasilianum* possessed similar or almost indistinguishable genetic background^38,40,41^. To gain further insight into sequence diversity in *PmMSP1*, the previously reported partial sequences of *PmMSP1* and the *MSP1* sequences of *P. brasilianum* (*PbrMSP1*) were included for comparison between the corresponding regions^38,39^. Despite short indels in block II, nine variants were identified. Of these, six variants occurred in Thai isolates whereas five variants were found in *PbrMSP1* in which alleles V and VI were shared between *PmMSP1* and *PbrMSP1* (Table 1).

**Table 1:** Distribution of alleles in block II of *PmMSP1* and *PbrMSP1*.

Allele	Sequence	Total	Distribution		
			Species		Country* (n)
			P.m	P.br	
I	NKDGNT––TTN–--ANN	21	21	–	Thailand (21)
II	NKDGNTSTTTN––-ANN	8	8	–	Thailand (8)
III	NKDGNT––TTNANNANN	1	1	–	Thailand (1)
IV	NKDGNTS-TTNANNANN	1	1	–	Thailand (1)
V	NKDGNT––TTD–-–ANN	9	7	**2**	Myanmar (3), Thailand (2), Cameroon (1), French Guiana (1) **French Guiana (2)**
VI	––––-–––-TN––-ANN	7	3	**4**	Thailand (2), Saudi Arabia (1), **Brazil (4)**
VII	––––-–––-TNANN––N	2	–	**2**	**Brazil (2)**
VIII	––––-–––-TDANN––N	1	–	**1**	**Brazil (1)**
IX	NKDGST––TTD–-–ANN	1	–	**1**	**French Guiana (1)**

P.m and P.br denote *Plasmodium malariae* and *P. brasilianum*, respectively. Thai isolates and GenBank accession numbers of isolates elsewhere are (I) PM2-PM8, PM10, PM14, PM16-PM19, PM22-PM26 and PM31-PM33; (II) PM9, PM11, PM12, PM21, PM28-PM30 and PM34; (III) PM1, (IV) PM35, (V) PM20, PM27, FJ824669, KX672046-KX672048, AF138879, AF138881 and AF138882; (VI) PM13, PM15, AF138878, FLQW01000468, KC906711, KC906714, KC906715, (VII) KC906713 and KC906716; (VIII) KC906712 and (IX) AF138880. *Bolds are countries for *P. brasilianum.*

### Diversity of block IV in *PmMSP1*

Of 32 codons in block IV of *PmMSP1*, amino acid substitutions were found in 20 residues, resulting in 16 haplotypes based on analysis of isolates from Thailand and elsewhere including those belonging to *PbrMSP1* (Table 2). Of these, 10 haplotypes were identified among Thai isolates in which three haplotypes were shared across endemic countries. All haplotypes of available *PbrMSP1* sequences (n = 4) were distinct from those of *PmMSP1*. However, allele VI from four Brazilian isolates (GenBank accession nos. KR072269, KR072272, KR072278 and KR072279) and allele XV from a Peruvian *Saimiri* monkey (KR072284) were closely related with a single amino acid difference (I233K)^38^. It is noteworthy that seven of nine amino acid substitutions in block IV of *PbrMSP1* were shared with those of *PmMSP1* (Table 2).

**Table 2:** Distribution of alleles in block IV of *PmMSP1* and *PbrMSP1*.

Allele	Sequence	Total	Distribution		
			Species		Country* (n)
			P.m	P.br	
I	YDNIATTNKELEAPSGSGSDDEDIKNCDKKQK	14	14		Thailand (11), Brazil (3)
II	......K......H.............A...E	8	8		Thailand (8)
III	.N...DE..K.....E...........NE...	6	6		Myanmar (3), Thailand (2), Cameroon (1)
IV	.............S..................	5	5		Thailand (5)
V	....T...N..VTSNVPRP.....T.......	5	5		Thailand (4), Saudi Arabia (1)
VI	........N..K.L.E.RP.....T.......	4	4		Brazil (4)
VII	........N..K...EPRP.....T.......	3	3		Brazil (3)
VIII	........N..K...E.RP.....R.......	1	1		Brazil (1)
IX	......K....................A...E	1	1		Thailand (1)
X	H.......N....S............Y.....	1	1		Thailand (1)
XI	........N..K...E.RP.....T.......	1	1		Thailand (1)
XII	.N...DE..K.....E................	1	1		Thailand (1)
XIII	.N...DE..K...S.E...........GI...	1	1		Thailand (1)
XIV	........N..K.H.E.RT.....T.......	2		**2**	**Brazil (2)**
XV	........N..K.L.E.RP....KT.......	1		**1**	**Peru (1)**
XVI	........N..K.H.E.RT.....T.Y.....	1		**1**	**Brazil (1)**

P.m and P.br denote *Plasmodium malariae* and *P. brasilianum*, respectively. Dots represent corresponding identical amino acids per allele I. Thai isolates and GenBank accession numbers of isolates elsewhere are (I) PM3-PM8, PM16-PM19, PM33, KR072273, KR072274 and KR072276; (II) PM13 and PM20-PM26; (III) PM1, PM27, KX672046–KX672048 and FJ824669; (IV) PM11, PM12 and PM28–PM30; (V) PM14, PM31, PM32, PM34 and FLQW01000468; (VI) KR072269, KR072272, KR072278 and KR072279; (VII) KR072271, KR072275 and KR072277; (VIII) KR072270; (IX) PM9; (X) PM10; (XI) PM15; (XII) PM2; (XIII) PM35; (XIV) KR072281 and KR072283; (XV) KR072284 and (XVI) KR072282. *Bolds are countries for *P. brasilianum.*

### Diversity of repeats in *PmMSP1*

Block VI contained complex repeat motifs with multiple patterns of repeat arrays and arrangements. Together with previously reported sequences of *PmMSP1* (n = 16) and *PbrMSP1* (n = 5), 35 haplotypes have been identified in this block in which 19 haplotypes occurred among Thai isolates (Fig. 3). The N-terminal part of this block contains non-repetitive amino acid sequences with variable indels, resulting in eight to 40 residues in this region. On the basis of distinct repeats and arrangements, block VI could be classified into types A and B. Type A contained 17 alleles (A1.1–A1.17) whereas type B could be further subdivided into subtypes B1 and B2, containing eight and 10 alleles, respectively (Fig. 3). Interestingly, the amino acid sequence of allele A1.10 was shared between *P. malariae* from a Brazilian patient (KR072216) and *P. brasilianum* from a Peruvian *Saimiri* monkey (JX045641) whereas the remaining *PbrMSP1* and most other *PmMSP1* type A alleles seemed to be closely related. It is noteworthy that none of *PbrMSP1* sequences belonged to type B. Meanwhile, the other repeat-containing region was located in block VIII spanning codons 989 and 1032 (residues after FJ824669), characterized by a degenerate octapeptide repeat motif, P(A)Q(T)P(S, T or Q)QA(S)A(S or T)L(S or V)P(V or -), with variation in the number of repeat units among isolates. Of 35 Thai isolates and 14 previously reported sequences, 13 haplotypes were identified in this block in which haplotype I was most common and occurred in isolates from Thailand, Myanmar and Brazil, followed by haplotype XIII which was shared between *PmMSP1* and *PbrMSP1*^35,38,39^ (Table 3).

**Table 3:** Distribution of alleles in block VIII of *PmMSP1* and *PbrMSP1*.

Allele	Sequence	Distribution (n)		
		Species		Country*
		P.m	P.br	
I	PQPQAALPAQPQAALPAQPQAALPAQPQAAVPAQSQATVPAQSQAAVPATTQSSSVSAPT	15		Thailand (12), Myanmar (1), Brazil (2)
II	PQPQAALPAQPQAALPAQPQAAV----------------PAQSQAAVPATTQSSSVSAPT	6		Thailand (6)
III	PQQQSSS-AQPQAALPAQPQAAV--------PAQSQATVPAQSQAAVPATTQSSSVSAPT	5		Thailand (5)
IV	PQPQAALPAQPQAALPAQPQAALPAQPQAALPAQPQAAVPAQSQAAVPATT---------	4		Thailand (4)
V	PQQQSSS-AQPQAAVPAQSQATV----------------PAQSQAAVPATTQSSSVSAPT	4		Thailand (3), Saudi Arabia (1)
VI	PQPQAALPAQPQAALPAQPQAAVPAQSQATV--------PAQSQAAVPATTQSSSVSAPT	2		Thailand (2)
VII	PQPQAALPAQPQAAVPAQSQATV----------------PAQSQAAVPATTQSSSVSAPT	1		Cameroon (1)
VIII	PQPQAALPAQPQAALPAQPQAALPAQPQAALPAQPQAAVPAQSQAAVPATTQSSSVSAPT	1		Thailand (1)
IX	PQQQSSS-AQPQAALPAQPQAAVPAQSQATVPAQSQAAVPAQSQAAVPATTQSSSVSAPT	1		Thailand (1)
X	PQPQAALPAQPQAAV––––––––––––––––––––––––––––––––PATTQSSSVSAPT	1		Thailand (1)
XI	PQPQAALPAQPQAALPAQPQAALPAQPQAAVPAQSQATVPAQSQAAVPATTQSSSVSAPT	1		Myanmar (1)
XII	PQPQAALPAQPQAALPAQPQAAVPAQSQAAL––––––––PAQSQAAVPATTQSSSVSAPT	1		Brazil (1)
XIII	PQPQAALPAQPQAALPAQPQAALPAQPQAAVPAQSQAALPAQSQAAVPATTQSSSVSAPT	8	**4**	Brazil (8), **Brazil (3)**, **Peru (1)**

P.m and P.br denote *Plasmodium malariae* and *P. brasilianum*, respectively. Dash indicates a deletion. Thai isolates and GenBank accession numbers of isolates elsewhere are (I) PM1, PM2, PM10, PM14, PM20–27; KX672048, KR072259 and KR072258; (II) PM3–PM7; (III) PM11, PM12 and PM28–PM30; (IV) PM16–PM19; (V) PM31, PM34, PM35 and FLQW01000468, (VI) PM32 and PM33; (VII) FJ824669; (VIII) PM9; (IX) PM13; (X) PM15; (XI) KX672047; (XII) KR072262; (XIII) KR072254–KR072257, KR072260–KR072263, KR072265–KR072268 and KY189272. *Bolds are countries for *P. brasilianum*.

### Microheterogeneity in conserved blocks

The complete sequences of all 5 conserved blocks have been available from 35 Thai isolates and an isolate from Cameroon (FJ824669). All nucleotide substitutions in conserved blocks were dimorphic, i.e. either one or the other of any two bases occurred at given positions. In total, 37 mutations were observed in conserved blocks, resulting in three haplotypes in blocks I and III, nine in block V, four in block VII and 13 in block IX (Table 4). The levels of nucleotide diversity in conserved regions ranged from 0.00098 to 0.00232 in blocks I and VII, respectively, which was an order or two orders of magnitude less than those in variable blocks (blocks IV, VI and VIII). Since the 19-kDa fragment of PfMSP1 has been considered to be an asexual blood stage vaccine target^26^, microheterogeneity in this region is of concern for vaccine development. Analysis of the homologous region to the 19-kDa-fragment-encoding sequence in *PmMSP1* has revealed 4 nucleotide substitutions: c.5045G>A (G1681E), c.5055A>C (E1684D), c.5060A>T (E1686V) and c.5074C>A (Q1691K) (positions after the FJ824669 sequence). In total, four haplotypes occurred in the putative 19-kDa fragment of PmMSP1, characterized by (1) G-E-E-Q, (2) E-E-E-K, (3) E-D-E-K and (4) E-E-V-K, in which haplotype I was found in the Cameroonian isolate (FJ824669) whereas the remaining haplotypes co-existed among *P. malariae* populations in Thailand. Of these four substituted residues, c.5044G>C+5045G>A (G1681Q) was found in three isolates from Brazil and constituted another haplotype characterized by Q-E-E-K although singletons were previously observed in other five positions of this region^38^.

**Table 4:** Haplotype and nucleotide diversity in the complete *PmMSP1* sequences.

Block	No. codons	M	S	H	*h* ± S.D	*π* ± S.E	*d*_S_ ± S.E	*d*_N_ ± S.E
I (conserved)	56	2	2	3	0.160 ± 0.080	0.00098 ± 0.00071	0.01980 ± 0.02021	0.00501 ± 0.00503
II (indels)	5–15	–	–	6	0.157 ± 0.077	–	0.00000 ± 0.00000	0.03810 ± 0.03041
III (conserved)	140	2	2	3	0.398 ± 0.081	0.00103 ± 0.00090	0.00000 ± 0.00000	0.00392 ± 0.00271
IV (variable)	32	30	27	10	0.838 ± 0.036	0.07763 ± 0.01894	0.01882 ± 0.01386	0.12613 ± 0.02543**
V (conserved)	440	10	10	9	0.740 ± 0.048	0.00144 ± 0.00061	0.00158 ± 0.00149	0.00397 ± 0.00130
VI (repeats)	97–229	–	–	20	0.863 ± 0.040	0.12162 ± 0.01382	–	–
VII (conserved)	156	10	10	4	0.340 ± 0.093	0.00232 ± 0.00088	0.01346 ± 0.00949	0.01282 ± 0.00454
VIII (repeats)	28–60	–	–	10	0.567 ± 0.071	0.02852 ± 0.00954	–	–
IX (conserved)	719	13	12	13	0.890 ± 0.027	0.00113 ± 0.00043	0.00115 ± 0.00110	0.00161 ± 0.00051
All	1696–1831	72	68	21	0.944 ± 0.021	0.01204 ± 0.00162	0.00208 ± 0.00077	0.00447 ± 0.00065*

*M* the number of mutations, *S* the number of segregating sites, *H* the number of haplotypes, *h *haplotype diversity, *π* nucleotide diversity, *d*_*S*_ number of synonymous substitutions per synonymous site, *d*_*N*_ number of nonsynonymous substitutions per nonsynonymous site, *S.D.* standard deviation, *S.E*. standard error. Analysis includes 35 Thai isolates and the FJ824669 sequence. Z-tests of the hypothesis that mean *d*_*S*_ equals that of mean *d*_*N*_: * *p* < 0.05; **p < 0.0005.

### Neutrality test

To test for departure from neutrality, nucleotide substitutions in nonrepeat regions were analyzed by comparing the rate of synonymous substitutions per synonymous site (*d*_S_) and that of nonsynonymous substitutions per nonsynonymous site (*d*_N_) for each block of *PmMSP1*. Results revealed that *d*_N_ exceeded *d*_S_ in conserved blocks II, V and IX, and variable block IV. However, significant difference between *d*_N_ and *d*_S_ was observed only in block IV (Z-test, *p* < 0.0005) (Table 4). Meanwhile, codon-based detection of deviation from neutrality by the FUBAR method has shown evidence of positive selection in blocks I (E26K), IV (P223L/S/H and K241E), V (P294R) and IX (P1045Q/R and E1684D). On the other hand, evidence of purifying selection was found at codon 1374 (GAT⟶GAC, p.D1374) in conserved block IX of PmMSP1, a homologous residue located in close proximity to the 38 kDa/42 kDa cleavage site in PfMSP1 (Supplemental Fig. S1).

### Recombination

Evidence of intragenic recombination in the *PmMSP1* gene was determined from 35 Thai isolates by using the RDP4 package which revealed 21 potential recombination sites across the coding region of this gene (Table 5). Recombination breakpoints were detected more commonly in repeats or variable blocks (28 of 42 sites, 66.7%) than in conserved blocks. On the other hand, no recombination event was detected in conserved blocks I, II and V. Recombination breakpoints spanned 41–3978 bp with an average length of 784 bp.

**Table 5:** Intragenic recombination in *PmMSP1* inferred from 35 Thai isolates.

Event no.	Recombination breakpoints				Methods (*p* value)						
	Between positions*		Between blocks		RDP	GENECONV	Bootscan	Maxchi	Chimaera	SiSscan	3Seq
1	2187	2728	VI	VII	4.69 × 10^–12^	1.07 × 10^–9^	1.72 × 10^–10^	7.26 × 10^–14^	1.93 × 10^–14^	1.79 × 10^–19^	2.03 × 10^–21^
2	2192	2841	VI	VII	3.02 × 10^–4^	4.76 × 10^–3^	NS	2.26 × 10^–5^	1.66 × 10^–6^	4.72 × 10^–11^	5.47 × 10^–4^
3	706	4684	IV	IX	NS	4.47 × 10^–5^	9.81 × 10^–6^	5.22 × 10^–5^	2.39 × 10^–5^	6.55 × 10^–5^	1.90 × 10^–9^
4	1556	4190	V	IX	NS	1.51 × 10^–3^	1.15 × 10^–4^	9.31 × 10^–5^	NS	9.78 × 10^–5^	1.19 × 10^–8^
5	2192	2353	VI	VI	4.47 × 10^–4^	NS	NS	3.65 × 10^–4^	5.06 × 10^–5^	8.60 × 10^–5^	8.78 × 10^–8^
6	1361	2979	V	VIII	1.87 × 10^–7^	6.47 × 10^–6^	NS	NS	NS	NS	NS
7	2146	2192	VI	VI	NS	0.02319	NS	6.99 × 10^–7^	9.12 × 10^–3^	NS	NS
8	2467	3057	VI	VIII	6.45 × 10^–5^	2.38 × 10^–5^	4.07 × 10^–6^	5.40 × 10^–3^	4.42 × 10^–3^	NS	2.97 × 10^–7^
9	2377	2462	VI	VI	NS	3.89 × 10^–5^	7.90 × 10^–6^	NS	NS	NS	9.50 × 10^–5^
10	2303	2369	VI	VI	NS	NS	NS	9.83 × 10^–4^	2.78 × 10^–3^	NS	1.08 × 10^–5^
11	644	1599	IV	V	NS	NS	NS	4.60 × 10^–3^	NS	NS	1.44 × 10^–5^
12	2482	2869	VII	VII	NS	6.08 × 10^–3^	2.19 × 10^–3^	NS	NS	NS	8.02 × 10^–5^
13	797	1844	V	V	NS	1.98 × 10^–2^	5.39 × 10^–3^	NS	NS	NS	8.63 × 10^–4^
14	2294	2466	VI	VI	NS	NS	NS	NS	NS	1.82 × 10^–13^	1.59 × 10^–3^
15	644	2147	IV	VI	NS	NS	NS	1.48 × 10^–2^	NS	NS	2.18 × 10^–3^
16	2197	2302	VI	VI	NS	NS	NS	NS	NS	NS	2.62 × 10^–3^
17	2212	2328	VI	VI	NS	NS	NS	NS	NS	NS	5.35 × 10^–3^
18	1847	2185	V	VI	8.05 × 10^–3^	NS	NS	NS	NS	NS	1.35 × 10^–2^
19	2357	2840	VI	VII	NS	NS	NS	NS	NS	NS	1.35 × 10^–2^
20	2172	2213	VI	VI	NS	NS	NS	2.18 × 10^–3^	NS	NS	2.63 × 10^–2^
21	644	1599	IV	V	NS	NS	NS	NS	NS	NS	2.73 × 10^–2^

*Positions after the FJ824669 sequence.

### Phylogenetic analysis

**Figure 4:**
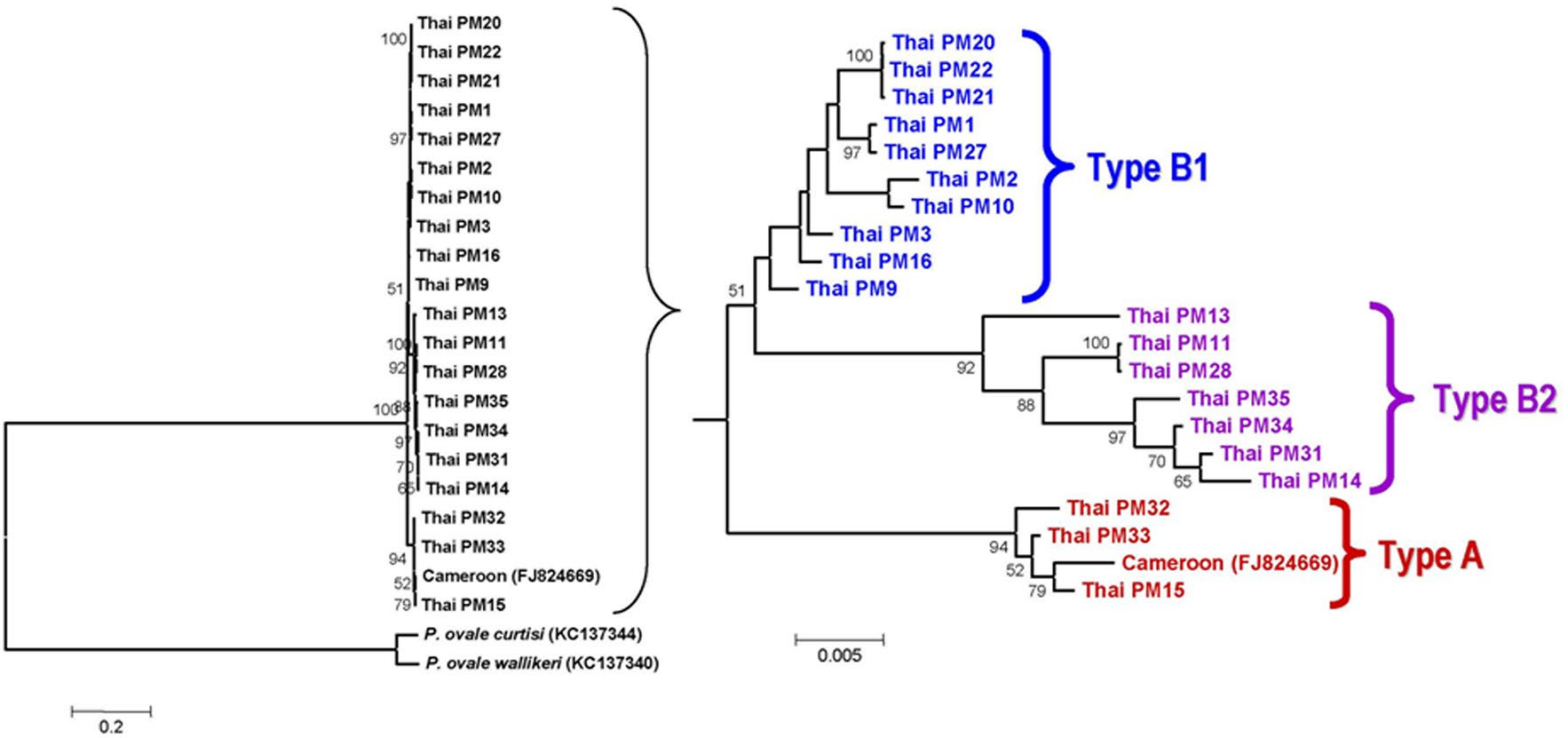
Maximum likelihood trees inferred from 21 distinct complete coding sequences of *PmMSP1* with *PocMSP1* and *PowMSP1* as outgroup sequences. Numbers on the branches are the percentage of 1000 bootstrap samples supporting the branch; only values greater than 50 are shown. Types are based on repeat classification in Fig. 3. Scale bar indicates nucleotide substitution per site.

Analysis of the complete coding sequences of *PmMSP1* has revealed two distinct clades in the phylogenetic tree (Fig. 4). The maximum likelihood tree inferred from the sequences of block VI per se has revealed 2 clades corresponding to characteristic repeats assigned to types A and B. It is noteworthy that the bifurcating clusters of taxa in the clade belonging to type B were in line with the isolates bearing types B1 and B2 repeats (Figs. 3, 4). On the other hand, the tree inferred from the sequences excluding block VI showed a different topology.

### Predicted linear B-cell epitopes

**Figure 5:**
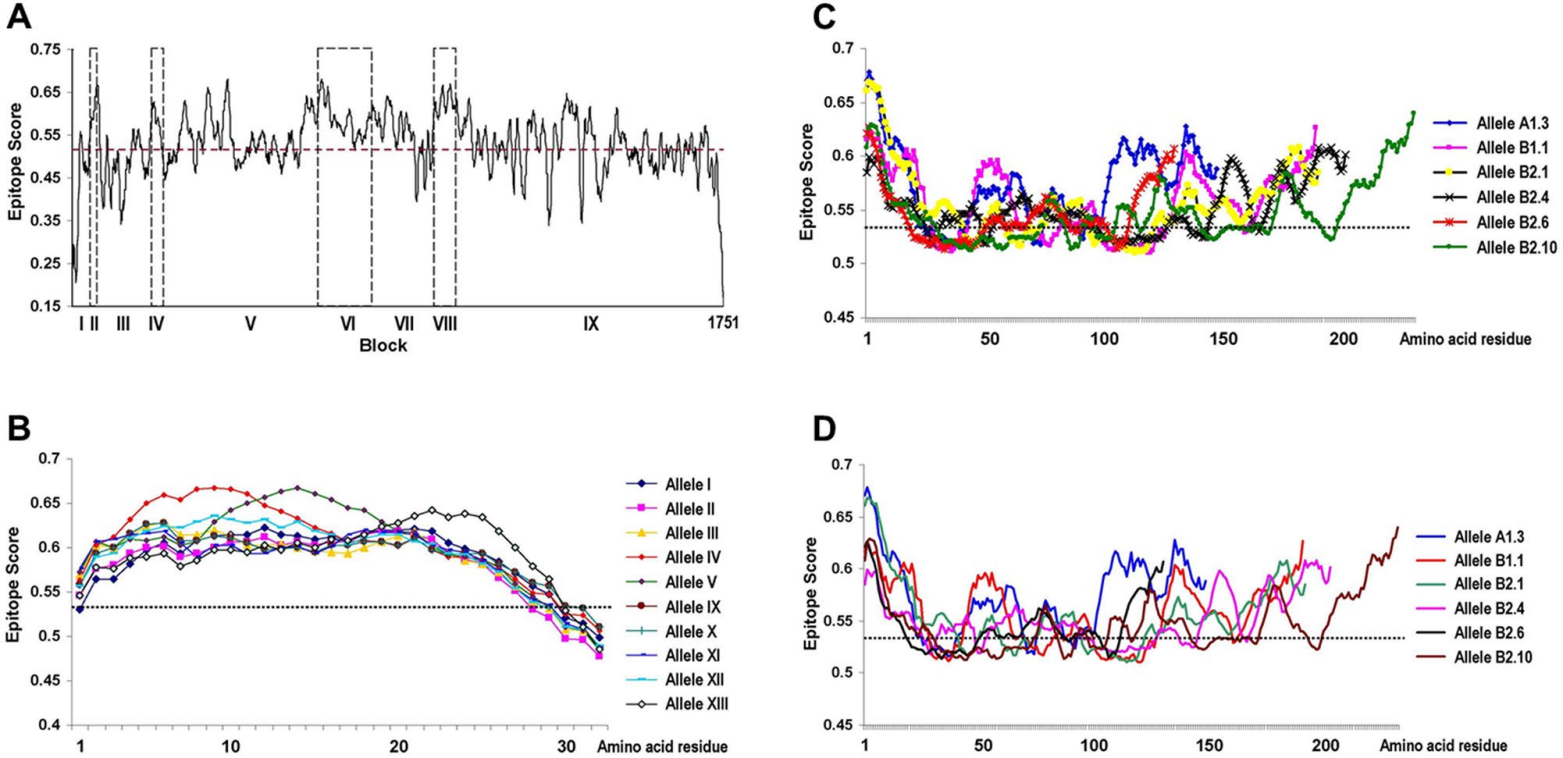
Predicted linear B-cell epitopes in PmMSP1 based on the BepiPred 2.0 method. (**A**) Epitope scores across the entire protein (GenBank accession no. FJ824669). Variable blocks are shown as broken boxes. (**B**–**D**) represent epitope scores for representative alleles of blocks IV, VI and VIII, respectively. The cutoff value is indicated by a broken line.

The graphical presentation from BepiPred 2.0 analysis has revealed a number of potential linear B-cell epitopes across PmMSP1, spanning both conserved and variable blocks (Fig. 5A). Short indels in block II did not affect predicted B-cell epitopes encompassing this region. Interestingly, amino acid substitutions in variable block IV seemed not to affect predicted linear B-cell epitopes in all variants (Fig. 5B). On the other hand, the predicted epitope scores were variable among different alleles of blocks VI and VIII (Fig. 5C,D). Variation in the predicted scores was more pronounced among variants in block VI in which some regions were below the cutoff threshold value for being linear B-cell epitopes.

### Predicted helper T-cell epitopes

Analysis of HLA-class II-binding peptides in PmMSP1 based on common HLA-DR alleles in Thai population (allele frequencies > 10%) including HLA-DRB1*12:02, -DRB1*15:02, -DQB1*05:01, -DQB1*05:02, -DQB1*03:01, -DQB1*03:03, -DQA1*01:01, -DQA1*01:02, -DQA1*03:02 and -DQA1*06:01 has predicted a number of potential binding peptides predominantly outside blocks VI and VIII which contained repeats (Supplemental Fig. S2). Block IV did not receive adequate scores for being HLA-class II-binding peptides (percentile rank < 10 and MHC binding affinity IC_50_ < 1000 nM) for these common HLA class II alleles^42,43^. However, searching for potential HLA-class II-binding peptides among alleles spanning block IV from residues 207 to 221 has shown that alleles II, IV and V had percentile ranks less than 10 and MHC binding affinity IC_50_ < 1000 nM for some uncommon HLA class II alleles in Thailand^44^. On the other hand, a potential HLA-class II-binding peptide was identified in one of nine alleles (allele V) of block IV encompassing residues 211–225 (Table 6). Taken together, these peptide variants could be potential helper T-cell epitopes in this molecule albeit being recognized by some uncommon HLA class II alleles among Thai population^44^.

**Table 6:** Predicted HLA class-II binding peptides in block IV of *PmMSP1*.

Amino acid residue†	Allele	Peptides and their variants	Prevalence (%, n = 35)	HLA§	Allele Frequency*	IC50#	Peptide rank#
207–221	I	KKEYNNIADENKKLE	11.43	None	–	–	–
	II	KKEY**D**NIA**TT**NK**E**LE	45.71	DQA1*04:01/ DQB1*04:02	0.0021/ 0.0032	747	9.9
	III	KKEY**D**NIA**TK**NK**E**LE	25.71	None	–	–	–
	IV	KKEY**D**NI**TTT**N**NE**L**V**	11.43	DRB1*13:02	0.0138	43.7	5.5
				DRB1*04:05	0.0489	235.4	10
	V	KKE**HD**NIA**TT**N**NE**LE	2.86	DQA1*04:01/ DQB1*04:02	0.0021/ 0.0032	478	6.4
				DQA1*03:01/ DQB1*03:02	0.0457/ 0.0426	878	9.9
	VI	KKEY**D**NIA**TT**N**NE**L**K**	2.86	None	–	–	–
211–225	I	NNIADENKKLEAPSE	8.57	None	–	–	–
	II	**D**NIA**TT**NK**E**LEAPS**G**	31.43	None	–	–	–
	III	**D**NIA**TK**NK**E**LEA**H**S**G**	22.86	None	–	–	–
	IV	**D**NIA**TT**NK**E**LEA**S**S**G**	14.29	None	–	–	–
	V	**D**NI**TTT**N**NE**L**VTSNV**	11.43	DRB1*13:02	0.0138	55.9	6.6
	VI	**D**NIA**TT**N**NE**LEA**S**S**G**	2.86	None	–	–	–
	VII	**D**NIA**TK**NK**E**LEAPS**G**	2.86	None	–	–	–
	VIII	**D**NIA**TT**N**NE**L**K**APSE	2.86	None	–	–	–
	IX	NNIADENKKLEA**S**SE	2.86	None	–	–	–

^**†**^Positions and amino acid substitutions are based on the FJ824669 sequence.

^§^Analysis based on the HLA alleles available in the IEDB analysis resource (accessed February 18, 2022).

*Allele frequency among Thai population^44^.

^#^Based on NN-align and the IEDB recommended 2.22 method^43^.

## Discussion

In this study, we have shown that the complete coding sequence of *PmMSP1*could be partitioned into five conserved and four variable blocks. Like other malarial *MSP1*s, conserved blocks of *PmMSP1* exhibited microheterogeneity of sequences with dimorphic nucleotide substitutions^24,32–34,45,46^. Comparative analysis has revealed that short indels in block II of *PmMSP1* seemed to be homologous to a short indel region at the 5′ portion of *PvMSP1*. Likewise, variable nonrepeat block IV of *PmMSP1* were found to be homologous to variable nonrepeat blocks of *PoMSP1,* and repeat domains of *PkMSP1* and *PvMSP1* (Supplemental Fig. 3). Likewise, repeat blocks VI and VIII of *PmMSP1* were homologous to blocks VIII and X of *PocMSP1* and *PowMSP1*, blocks IV and VI of *PkMSP1*, and blocks VI and VIII of *PvMSP1* (Supplemental Fig. 3). Meanwhile, the distantly related *PfMSP1* also contained repeats in blocks VIII homologous to block VI of *PmMSP1.* Although variable and semi-conserved blocks of *PfMSP1* consisted of two distinct parental alleles (MAD20 and K1), sequences of these regions were highly conserved within each allelic family^24,45^. Therefore, intraspecific conserved blocks of these malarial *MSP1* genes seemed to be largely found in corresponding locations. Taken together, the similarity in primary structural organization of *MSP1*s across *Plasmodium* species may suggest that this locus has evolved from a common ancestral sequence whereas the lack of homologous regions in some domains of the genes among species could imply post-speciation evolution of individual *MSP1* lineages. Consistently, it has been suggested that positive selection could influence lineage-specific evolutionary history of some human and simian malarial *MSP1*s^47^.

It is noteworthy that the levels of nucleotide diversity of *PkMSP1, PvMSP1* and *PfMSP1* were comparable among Thai isolates. On the other hand, the level of nucleotide diversity of *PmMSP1* was significantly less than those of *PkMSP1, PvMSP1* and *PfMSP1* but remarkably greater than those of *PoMSP1*^19,32,33,48–50^. Consistent findings were observed when analysis was performed separately for synonymous (π_S_) and nonsynonymous sites (π_N_) (Supplemental Table 2). The extent of nucleotide diversity among *MSP1*s of different *Plasmodium* species in Thailand could be due to evolutionary and population genetic forces on parasite populations such as mutation, recombination and population processes. Meanwhile, the neutral theory of molecular evolution predicts that the level of nucleotide diversity is proportional to the mutation rate (μ) and effective population size (*N*_e_) under mutation-drift equilibrium^51^. Since the mutation rates of malarial *MSP1* genes seemed to be similar across species^52–54^, variation in the levels of nucleotide diversity of these loci could be due to the difference in effective population sizes among *Plasmodium* species in Thailand. Although some regions or residues in malarial *MSP1*s were deviated from selective neutrality, the remaining majority of sequences seemed to be under neutral evolution. Therefore, the level of nucleotide diversity may roughly reflect the number of breeding individuals in the population. On the other hand, the low level of nucleotide diversity in *PmMSP1* could represent the low transmission rate and probably from bottleneck effects due to malaria control measures as previously noted^38,46^. Our previous surveys of malaria in Thailand have shown that the prevalence of *P. malariae* and *P. knowlesi* in Thailand was comparable^15,18–20^. Therefore, it is likely that the higher level of nucleotide diversity of *PkMSP1* than that of *PmMSP1* could stem from a hidden large reservoir of *P. knowlesi* in its macaque natural hosts in this country^33,55^. On the one hand, the haplotype diversity of most malarial *MSP1* genes in Thailand was relatively high (> 0.9), implying that distinct or rare haplotypes were abundant in the populations (Supplemental Table S2). On the other hand, we observed some predominant *PmMSP1* haplotypes in this country, i.e. haplotypes III, XIII and X, which occurred across endemic provinces and between long time intervals of sample collections (Fig. 1), suggesting that the parasites bearing these haplotypes could probably have reproductive advantage.

Conserved blocks in *PmMSP1* displayed microheterogeneity of sequences in which nucleotide substitutions seems to have evolved neutrally because block-wise analysis revealed that *d*_S_ was not significantly different from *d*_N_ (Table 4). However, codon-based analysis has identified four positively selected codons in conserved blocks, suggesting that natural selection has influenced evolution of particular codons. Interestingly, one of these codons (residue E1684D) was located between the two EGF-like domains at the C-terminal part of PmMSP1 in which the homologous region in PfMSP1 has been a target for naturally acquired antibodies associated with clinical protection against falciparum malaria^26^. Intriguingly, positive selection in the EGF-like domain of PmMSP1 could probably be driven by host immune pressure. On the other hand, evidence for purifying selection was detected at codon 1374 (GAT ⟶ GAC) that was located in close proximity to the canonical 38 kDa/42 kDa cleavage site in PfMSP1^56^. Importantly, cleavage at this site has been shown to be a rate-limiting processing step, suggesting its pivotal role for MSP1 proteolytic maturation^57,58^. Therefore, deviation from selective neutrality occurred at particular residues in conserved regions of PmMSP1.

The variable nonrepeat block IV of *PmMSP1* spanned 32 codons with 21 amino acid substitutions, resulting in 16 alleles among Thai and global isolates (Table 2). The significant difference in *d*_N_ exceeding *d*_S_ in this block implies that positive selection could influence diversity in this region (Table 4). On the basis of amino acid alignment, block IV of PmMSP1 was homologous to block III of PfMSP1, a portion of the 83-kDa fragment which forms a flexible wing domain of the protein as demonstrated by single-particle cryo-electron microscopy^31^. Several lines of evidence have suggested that MSP1 could be detected as monomeric and dimeric forms^58–60^. It has been shown that dimerization of PfMSP1 involves the interaction between the 83-kDa and 42-kDa fragments^31^. Although the significance of dimerization of PfMSP1 remains unknown, the protective capability against falciparum malaria conferred by natural antibodies to the 83-kDa fragment could suggest the functional importance of this region^61^. Importantly, in silico analysis has shown that block IV of PmMSP1 contained both B-cell and helper T-cell epitopes. Consistently, recombinant proteins derived from various regions of PmMSP1 including the N-terminal fragment elicited strong immunogenicity in mice^62^ and were highly recognized in serum samples of primates and non-human primates from malaria endemic areas^63,64^. Although allelic variation in block IV of this protein seemed not to drastically change the propensity of being B-cell epitopes as predicted by the IEDB analysis resource (Fig. 5B), amino acid substitutions in this region were unlikely recognized by common HLA class II alleles among Thai population (Fig. 4B, Table 6, Supplemental Fig. S2). Importantly, mutations in block IV may reduce or totally abolish predicted binding capability of the peptides to some uncommon HLA class II alleles in Thai population (Table 6; Supplemental Fig. S2). Undoubtedly, further studies are required to address the immunological significance of helper T-cell epitopes in block IV of PmMSP1. Therefore, it seemed that positive selection in block IV could probably be driven by host immune pressure.

Repetitive amino acid sequences have been observed in several malarial antigens including MSP1s. Our analysis has revealed two repeat-containing regions in blocks VI and VIII of PmMSP1. Unlike block VIII that contained degenerate octapeptide motifs, repeats in block VI were more complex, characterized by a repertoire of different repeat arrays and arrangements. Meanwhile, the RDP4 package has identified 21 recombination breakpoints in *PmMSP1*. Interestingly, about half of recombination events involved block VI whereas about one-third of the breakpoints occurred within this block. Besides slip-strand mispairing mechanism that could generate sequence and size variation in repeat sequences, recombination may contribute to shuffle of repeat units in block VI. Although a number of linear B-cell epitopes were predicted in this block (Fig. 5A), in silico analysis has suggested that variation in repeat sequences could affect antibody recognition (Fig. 5C). Meanwhile, phylogenetic tree inferred from the block VI sequences of *PmMSP1* has revealed two distinct clades, corresponding to repeat sequence types A and B of this block. Importantly, variation in the number of repeat units could affect intensity of antibody reactivity whereas distinct variants of repetitive antigens may abolish specific antibody response as shown by antibody recognition of repeat antigens in block II of PfMSP1^65,66^. Therefore, sequence divergence of repetitive regions in PmMSP1 could probably enhance host immune evasion by the parasites.

One of the shared features of PmMSP1 and PvMSP1 was the presence of indels near the N-terminus of the proteins. Although indels in block II spanned 17 codons, 6 alleles have been identified among Thai isolates (Table 1). Indels are commonly found in both coding and noncoding regions of prokaryotes and eukaryotes genomes while they may occur within repeats and nonrepeat regions^67,68^. The generation of indels related with repeats could be due to polymerase slippage^69,70^. On the other hand, the formation of indels in nonrepeat regions required pre-existing palindromic or quasi-palindromic sequences, provoking a double-stranded break intermediate during DNA replication while the ensuing repair process was imperfect^71–74^. It is noteworthy that quasi-palindromic repeats were identified around indels of *PmMSP1* and *PvMSP1*, supporting the mechanisms for indel formation in nonrepeats of these genes (Supplemental Fig. S4). Although analysis of natural selection on these indels was not possible due to unknown ancestral state of this region, the lack of frame-shift mutation following indels in both *PmMSP1* and *PvMSP1* could imply selective constraint on the protein structure and/or function.

Several lines of evidence have suggested that *P. malariae* and *P. brasilianum* were de facto either con-species or the same parasites^38,40,41^. A repertoire of alleles in block VI constituting the most polymorphic region of the gene has been identified among *PmMSP1* and *PbrMSP1* (Fig. 2; Table 4). Importantly, allele A1.10 of block VI was shared between the *MSP1* genes of *P. malariae* and *P. brasilianum* whereas allele XIII of block VIII has been previously reported to occur in both species^38^. Like other genes or non-coding loci containing repeats in malarial genomes, variation in repeat sequences and the number of repeat units could be generated by the process of slip-strand mispairing mechanism^75,76^. Therefore, it is unlikely that identical complex repeats could have arisen from homoplasy. Furthermore, shared alleles between *PmMSP1* and *PbrMSP1* have been observed in variable blocks II (alleles V and VI) and VIII (allele XIII) whereas a single codon difference was observed between alleles VI and XV in variable block IV (alleles VI and XV) (Tables 1, 2, 3). Taken together, it is likely that *P. malariae* and *P. brasilianum* could be identical species or at least con-species as previously noted^38,41^.

In conclusion, analysis of the complete coding sequences of *PmMSP1* from clinical isolates has revealed structural organization of this locus. Besides structural similarity across human malarial *MSP1*s, evidences of intragenic recombination and natural selection have been identified in *PmMSP1*. The information from this study could be useful for further studies such as vaccine development and strain characterization of *P. malariae* based on this molecule.

## Materials and methods

### Parasite isolates

Thirty-five *Plasmodium malariae* isolates were obtained from symptomatic malaria patients during surveys of *Plasmodium* species distribution in Thailand during 1994 and 2016 (Fig. 1). Either finger-pricked or venous blood samples were taken from each subject and spotted onto filter papers or preserved in EDTA, respectively. Both thin and thick blood films were prepared from fresh blood and stained with Giemsa solution for microscopic examination of malaria parasites. DNA was extracted from each blood sample using Qiagen DNA mini kit (Qiagen, Hilden, Germany) per the manufacturer’s recommendation and kept at − 40 °C until use. Definite species identification was performed by species-specific nested PCR targeting *18S rRNA,* mitochondrial *cytochrome b* or *cytochrome oxidase I* as previously described^15,19,20^.

### PCR amplification and sequencing of the *PmMSP1* gene

The complete coding sequence of *PmMSP1* was amplified by nested PCR using outer primers: Pmmsp1F0 (5′-TACTCTATATTATCAAGTTTAATTC-3′) and Pmmsp1R0 (5′-CATTCGTATCCTTCTTTTCTGT-3′), and inner primers: Pmmsp1F01 (5′-GTTTAATTCAAAAATGAAAGCAC-3′) and Pmmsp1R01 (5′-TCTTTTTTTCTTAAAGTAAGTTAAAC-3′). Amplification reaction and condition were as previously described^34^. All amplification reactions were done in an Applied Biosystem GeneAmpH PCR System 9700 thermocycler (PE Biosystems, Foster City, CA). PCR products were analyzed by 1% agarose gel electrophoresis. The PCR products were purified by using a QIAamp PCR purification kit (Qiagen) and used as templates for sequencing. Sequencing primers were deployed to obtain overlapping sequences of the gene in which both directions were determined directly from the PCR-purified templates (Supplemental Table S1). Validation of singletons and indels in the sequences was performed by sequencing of the PCR products from independent amplification reactions using the same genomic DNA as templates.

### Data analysis

Alignment of the *PmMSP1* nucleotide sequences was performed by using the default option of the MUSCLE program and manually edited^77^. Indels in coding regions were determined from multiple alignments of amino acid sequences to maintain the reading frame. The sequence of the first complete *PmMSP1* gene from a Cameroonian patient was used as reference (GenBank accession number FJ824669)^36^. Tandem repeats were analyzed by scanning each sequence using window sizes per the default option of the Tandem Repeats Finder version 4.0 algorithm^78^. Nucleotide diversity was computed from the average number of nucleotide differences per site between two sequences in the sample and the standard errors were estimated by 1000 boostrap pseudoreplicates^79^. A sliding window analysis of nucleotide diversity was performed by using window length of 100 nucleotides and step size of 15 sites. Haplotype diversity and its sampling variance were determined by using the DnaSP program^80^. The number of synonymous substitutions per synonymous site and the number of nonsynonymous substitutions per nonsynonymous site was computed using Nei and Gojobori’s method^79^ with Juke and Cantor correction^81^. Standard errors of these parameters were estimated by the bootstrap method with 1000 pseudoreplicates using the MEGA 6.0 program^82^. Differences between the nucleotide diversity values were determined by a two-tailed Z-test. Deviation from selective neutrality at individual codons was identified using fast unconstrained Bayesian approximation (FUBAR) method implemented in the Datamonkey Web-Server^83,84^. To minimize the interfering signals from recombination on selection of individual codons, the data generated by elimination of recombination segments was deployed for analysis^85^. Determination of intragenic recombination was performed by using the Recombination Detection Program version 4 (RDP4)^86^. Phylogenetic trees were constructed by using the Maximum Likelihood method based on the General Time Reversible model with a discrete Gamma distribution to model evolutionary rate differences among sites^84^. The final tree is the one with the highest log likelihood value. Bootstrap supports for the branching patterns were estimated from 1000 pseudoreplicates of the sample data. Prediction of linear B-cell epitopes was done by using the BepiPred linear epitope prediction 2.0 implemented in the Immune Epitope Database (IEDB) And Analysis Resource^87^. The threshold for linear B-cell epitopes was more than or equal to the average predicted residue score of the protein. The HLA-class II-binding peptides were predicted by using the IEDB recommended 2.22 algorithm with a default 12–18 residues option^88^. The criterion for being HLA-class II-binding peptides included the percentile rank ≤ 10 and the IC_50_ threshold for MHC binding affinity ≤ 1000 nM^43^. The common HLA class II haplotypes among Thai population were based on allele frequency ≥ 0.1 according to the previous report^44^.

### Ethical approval

The study protocol was approved by the Institutional Review Board on Human Research of Faculty of Medicine, Chulalongkorn University (IRB No. 384/60 and COA No. 805/2018). Written informed consent was obtained from participants or from parents or guardians prior to blood sample collections. All procedures were performed in accordance to the relevant guidelines and regulations.

## Supplementary Information

Supplementary Information.

## Data Availability

Thirty-five complete coding sequences of *PmMSP1* have been deposited in NCBI GenBank under accession numbers OM525734–OM525768. The datasets generated during and/or analyses during the current study are available from the corresponding author upon request.
