# Supplementary material for: Structural organization and sequence diversity of the complete nucleotide sequence encoding the Plasmodium malariae merozoite surface protein-1
Source: Sci Rep. 2022 Sep 16;12:15591. doi: 10.1038/s41598-022-19049-z (PMC9481586; doi:10.1038/s41598-022-19049-z)
Supplement: Supplementary file 1 — Supplementary Information. [file 41598_2022_19049_MOESM1_ESM.pdf]

Structural organization and sequence diversity of the complete nucleotide sequence encoding the *Plasmodium malariae* merozoite surface protein-1

Chaturong Putaporntip[1,]*, Napaporn Kuamsab[1,2], Rattanaporn Rojrung[1], Sunee Seethamchai[3], Somchai Jongwutiwes[1,]**

[1]Molecular Biology of Malaria and Opportunistic Parasites Research Unit, Department of Parasitology, Faculty of Medicine, Chulalongkorn University, Bangkok; [2]Cannabis Health Sciences, College of Allied Health Sciences, Suan Sunandha Rajabhat University, Samut Songkhram Province; [3]Department of Biology, Faculty of Science, Naresuan University, Pitsanulok Province, Thailand.

SUPPLEMENTAL INFORMATION

Contents

Table S1. Sequencing primers for *PmMSP1*…..……………………………………..…...…...….2

Table S2. Haplotype and nucleotide diversity in the *MSP1* genes of *P. malariae*, *P. ovale curtisi*, *P. ovale wallikeri*, *P. knowlesi*, *P. vivax* and *P. falciparum* from diverse endemic areas of Thailand…………………………………………….……………..…....3

Figure S1. Amino acid substitutions in the putative 42 KDa- and 19 KDa-fragments of PmMSP1 relative to the two parental strains of PfMSP1 (MAD20 and K1)……….4

Figure S2. Predicted common Thai HLA-class II-binding peptides in PmMSP1………..…….5

Figure S3. Schematic representation of malarial MSP1s, depicting homologous regions……..9

Figure S4. Quasi-palindromic sequences around the insertion/deletion region at the 3' portion of *PmMSP1* and *PvMSP1*…………………..……………………..……10

Supplemental Table S1 Sequencing primers for *PmMSP1*

Primer Name	Sequence (5'→ 3')	Direction	Positions after GenBank no. FJ824669
PmMSP1-F1	CTATATTATCAAGTTTAATTCAAA	Forward	(-25) – (-2)
PmMSP1-F2	TTGCTCGACGAAATAAAGGAAATTG	Forward	844 - 868
PmMSP1-F3	CGGAACAGGAGAACAATCCGAAC	Forward	1863 - 1885
PmMSP1-F4	GAAACTCAGGAAATTGGAATCGTT	Forward	2515 - 2538
PmMSP1-F5	ACAAGCAGTACTAATCATATTAAAG	Forward	3436 - 3460
PmMSP1-F6	TCAATTTAAACATGCAACTTC	Forward	4350 - 4370
PmMSP1-R1	ATCCTTCTTTTCTGTCTTTTTTTC	Reverse	1 - 24
PmMSP1-R2	TCAACATCTAATAGTTTATACG	Reverse	4394 - 4415
PmMSP1-R3	ATTTTCAACTTCTTTCTTTTCTGC	Reverse	3565 - 3588
PmMSP1-R4	TTGATATGACACACATAAGCAGT	Reverse	2593 - 2615
PmMSP1-R5	CTTGATTTTTTCTTCCTCCAGTTTTTTC	Reverse	1968 - 1995
PmMSP1-R6	ATCTGTAAATAGACCATCCAT	Reverse	1006 - 1026

Supplemental Table S2 Haplotype and nucleotide diversity in the *MSP1* genes of *P. malariae*, *P. ovale curtisi*, *P. ovale wallikeri*, *P. knowlesi*, *P. vivax* and *P. falciparum* from diverse endemic areas of Thailand

Gene	n	H	$h \pm$ S.D.	$\pi \pm$ S.E.	$\pi_S \pm$ S.E.	$\pi_N \pm$ S.E.	References
PmMSP1	35	20	0.941 ± 0.021	0.01135 ± 0.00136	0.01014 ± 0.00151	0.01166 ± 0.00116	This study
PocMSP1	5	3	0.700 ± 0.218	0.00129 ± 0.00040	0.00124 ± 0.00081	0.00129 ± 0.00038	1
PowMSP1	5	4	0.900 ± 0.161	0.00024 ± 0.00015	0.00074 ± 0.00056	0.00010 ± 0.00010	1
PkMSP1	12	10	0.970 ± 0.044	0.04532 ± 0.00251	0.05475 ± 0.00442	0.04251 ± 0.00207	2
PvMSP1	207	108	0.930 ± 0.015	0.04842 ± 0.00172	0.05699 ± 0.00423	0.04594 ± 0.00245	3, 4
PfMSP1	80	24	0.890 ± 0.025	0.04569 ± 0.00699	0.05139 ± 0.00303	0.04192 ± 0.00161	5-7

n, number of taxon; H, number of haplotypes; *h*, haplotype diversity; π, nucleotide diversity, π_S , nucleotide diversity at synonymous site; and π_N, nucleotide diversity at nonsynonymous site.

Z-tests of the hypothesis that π, π_S or π_N in *PmMSP1* equals the corresponding value in *PocMSP1, PocMSP1, PkMSP1, PvMSP1* or *PfMSP1*: all *p* values < 0.00001.

Z-tests of the hypothesis that π, π_S or π_N in *PkMSP1* equals the corresponding value in *PvMSP1* or *PfMSP1*: all *p* values > 0.05.

References:

1. Putaporntip, C., Hughes, A.L. & Jongwutiwes, S. Low level of sequence diversity at merozoite surface protein-1 locus of *Plasmodium ovale curtisi* and *P. ovale wallikeri* from Thai isolates. *PLoS One* **8**, e58962 (2013).
2. Putaporntip, C., Thongaree, S. & Jongwutiwes, S. Differential sequence diversity at merozoite surface protein-1 locus of *Plasmodium knowlesi* from humans and macaques in Thailand. *Infect. Genet. Evol.* **18**, 213-9 (2013).
3. Putaporntip, C. *et al*. Mosaic organization and heterogeneity in frequency of allelic recombination of the *Plasmodium vivax* merozoite surface protein-1 locus. *Proc. Natl. Acad. Sci. USA.* **99**, 16348-16353 (2002).
4. Jongwutiwes, S., Putaporntip, C. & Hughes, A.L. Bottleneck effects on vaccine-candidate antigen diversity of malaria parasites in Thailand. *Vaccine* **28**, 3112-7 (2010).
5. Tanabe, K. *et al*. Allelic dimorphism-associated restriction of recombination in *Plasmodium falciparum msp1*. *Gene* **397**, 153-160 (2007).
6. Tanabe, K. *et al*. Within-population genetic diversity of *Plasmodium falciparum* vaccine candidate antigens reveals geographic distance from a Central sub-Saharan African origin. *Vaccine* **31**, 1334-1339 (2013).
7. Tanabe, K. *et al*. *Plasmodium falciparum*: Genetic diversity and complexity of infections in a isolated village in Western Thailand. *Parasitol. Int.* **64**, 260-266 (2015).

Supplemental Figure S1. Amino acid substitutions in the putative 42 KDa- and 19 KDa-fragments of PmMSP1 relative to the two parental strains of PfMSP1 (MAD20 and K1).

```
PfMSP1-MAD20    NKKVNEALKS YENFLPEAK- VTTVVTPPQP DVTPSPLSVR VSGSSGSTKE ETQIPTSGSL LTELQQVVQL QNYDEEDDSL VVLPIFGESE DNDEYLDQVV  1304
PfMSP1-K1       NTDVNNALES YKKFLPEGTD VATVVSE--- ---------- -SGSDTLEQS QPKKPASTHV GAESNTITTS QNVDDEVDDV IIVPIFGESE EDYDDLGQVV  1251
PmMSP1          NEEVNKALNV YEELLPK--- --QISTEEQP D------NAL ADGTENATEG AEVRAATAES LVQGE----- DEYPEEVDEV IVFPIVGKKE KE-NPLDQIT  1377
  variants      .......... .......--- --....K... .------... .......... .......... ....------ .......... .......... ..-.......

                  ← 42 KDa fragment
PfMSP1-MAD20    TGEAISVT-M DNILSGFENE YDVIYLKPLA GVYRSLKKQI EKNIITFNLN LNDILNSRLK KRKYFLDVLE SDLMQFKHIS SNEYIIEDSF KLLNSEQKNT  1403
PfMSP1-K1       TGEAVTPSVI DNILSKIENE YEVLYLKPLA GVYRSLKKQL ENNVMTFNVN VKDILNSRFN KRENFKNVLE SDLIPYKDLT SSNYVVKDPY KFLNKEKRDK  1351
PmMSP1          KGQAETKQ-D DNILKPITNE YEVLYIKPLA GVYRVLRKQI GDQIDAFNSN LTNALDTRKK KRTYFLDVLN SDLIQFKHAT SDSYIIKDPY KLLDVDKKAK  1476
  variants      ........-. .......... .......... .......... .......... .......... .......... .......... .......... ..........

PfMSP1-MAD20    LLKSYKYIKE SVENDIKFAQ EGISYYEKVL AKYKDDLESI KKVIKEEKEK FPSSPPTTPP SPAKTDEQKK ESKFLPFLTN IETLYNNLVN KIDDYLINLK  1503
K PfMSP1-K1     FLSSYNYIKD SIDTDINFAN DVLGYYKILS EKYKSDLDSI KKYI------ ---------- ----NDKQGE NEKYLPFLNN IETLYKTVND KIDLFVIHLE  1431
PmMSP1          LIGSYKYIVS AIEKDITSAE NGVEYYDKMT KLYKTQLEAV KSAIAEAQKE ---------- ----GDKKTE NEKYIPFLTN MQTLYENLLN KINGNIINLK  1562
  variants      .......... .......... K......... .......... .......... ---------- ----...... .......... .......... ..........

                                                                                                         ← 19 KDa fragment
PfMSP1-MAD20    AKINDCNVEK DEAHVKITKL SDLKAIDDKI DLFKNTNDFE AIKKLINDDT KKDMLGKLLS TGLV-QNFPN TIISKLIEGK FQDMLNIS-Q HQCVKKQCPE  1601
PfMSP1-K1       AKVLNYTYEK SNVEVKIKEL NYLKTIQDKL ADFKKNNNFV GIADLSTDYN HNNLLTKFLS TGMVFENLAK TVLSNLLDGN LQGMLNIS-Q HQCVKKQCPQ  1530
PmMSP1          TLITNCNLEK DAVNITISKL TEYSKFDEKI EMFKNSKN-- -----EKDIA SSGILDILKQ KGLVNKNEST KIISELLGVD SNALLNISAK HACTETKYPE  1655
  variants      .......... .......... .D........ ........-- -----..... .......... .......... .......... .......... ..........

PfMSP1-MAD20    NSGCFRHLDE REECKCLLNY KQEGDKCVEN PNPTCNENNG GCDADATCTE EDSGSSRKKI TCECTKPDSY PLFDGIFCSS SNFLGISFLL ILMLILYSFI  1701
PfMSP1-K1       NSGCFRHLDE REECKCLLNY KQEGDKCVEN PNPTCNENNG GCDADAKCTE EDSGSNGKKI TCECTKPDSY PLFDGIFCSS SNFLGISFLL ILMLILYSFI  1630
PmMSP1          NAGCYRYEDG KEVWRCLLNY KLVDGGCVED EEPSCQVNNG GCAPEANCTK GDD----NKI VCACNAPYSE PIFEGVFCGS SSFLGLSLLL AALLIMFNLL  1751
  variants      .......... .......... .....E..D. V....K.... .......... ...----... .......... .......... .......... ..........
```

Note: GenBank accession numbers are X05624, X03371 and FJ824669 for PfMSP1 (MAD20 and K1 strains) and PmMSP1 (Cameroon strain). Variants include 35 Thai PmMSP1 sequences (GenBank accession nos. OM525734- OM525768). Dots represent identical residues with PmMSP1 (FJ82669). Dashes are deletions. Cysteine residues forming epidermal growth factor (EGF)-like domains 1 and 2 are highlighted in red and blue residues, respectively. Positively and negatively selected amino acids are highlighted in black and green, respectively. Amino acid positions are shown on the right.

Supplemental Figure S2. Predicted common Thai HLA-class II-binding peptides in PmMSP1 (GenBank accession no. FJ824669). Epitopes are marked with asterisks underneath amino acid residues. #Common HLA-class II alleles among Thai population are after reference 44.

```
HLA#                  ← Block I                                           →← Block II→← Block III
                      MKALIFLFSF VFFSINCHCE TNEDYEQLIQ KLGKLEELVV EGYNLFHKKK FALTDINKDG NTSTTDANNK DDSKVSSVTA KIGNFVSKVL NLNLPGYVQL TFSIRELITK    110
DRB1*12:02                                     ******* ********** ********** *******                                           ***** **********
DRB1*15:02            ********** ********** ****             **** ********** *******                     ***** ********** ******
DQA1*01:01/DQB1*03:01                                                                                                 ** ********** ****
DQA1*01:01/DQB1*03:03
DQA1*01:02/DQB1*03:01
DQA1*01:02/DQB1*03:03
DQA1*03:02/DQB1*03:01
DQA1*03:02/DQB1*03:03
DQA1*06:01/DQB1*03:01
DQA1*06:01/DQB1*03:03
DQA1*06:01/DQB1*05:01                                                                                                    ******** **********
DQA1*06:01/DQB1*05:02                                                                                                     ******* **********

                                                                                                                  →← Block IV
                      YSGLKYLIEG YEEFNELMYG INFYYDLLRA KLNDMHLNGY CDIPNHLKIN EKELEMLKKV VFGYRKPLEN IKDDINKMEK FITTNEATVN NIKELIKKEY NNIADENKKL    220
DRB1*12:02            ********** *      *** ********** *******          *** ********** ********** *                **** ********** ***
DRB1*15:02                         ******** ********** *****
DQA1*01:01/DQB1*03:01
DQA1*01:01/DQB1*03:03
DQA1*01:02/DQB1*03:01
DQA1*01:02/DQB1*03:03
DQA1*03:02/DQB1*03:01
DQA1*03:02/DQB1*03:03
DQA1*06:01/DQB1*03:01
DQA1*06:01/DQB1*03:03
DQA1*06:01/DQB1*05:01 **                ******** ********** **
DQA1*06:01/DQB1*05:02 *                  ******* ********** ***

                                         →← Block V
                      EAPSESGSDD EDIKNCNEKQ KIYKSRYNIL FYEKQLLEAQ KLIEVLKKRI QTLKENTDIK KLLDEIKEIE GKLPTSGSEA SASAAAPGAI KEPENTQIKE RQEKIKEIAK    330
DRB1*12:02                              *** ********** ********** ********** *******
DRB1*15:02                             **** ********** ********** ***                                                               ****
DQA1*01:01/DQB1*03:01                                                                           ** ********** ********** *****
DQA1*01:01/DQB1*03:03
DQA1*01:02/DQB1*03:01                                                                           ** ********** ********** *****
DQA1*01:02/DQB1*03:03                                                                            * ********** ********** ****
DQA1*03:02/DQB1*03:01                                                                           ** ********** ********** ******
DQA1*03:02/DQB1*03:03                                                                            * ********** ********** *****
DQA1*06:01/DQB1*03:01                                                                           ** ********** ********** ******
DQA1*06:01/DQB1*03:03                                                                            * ********** ********** *****
DQA1*06:01/DQB1*05:01                                                                                                              *****
DQA1*06:01/DQB1*05:02

                      NIVFNMDGLF TDAFELDYYV REKEKKSFNS ATTQLANGKA VNRTPPAPVM YPHGIIYAVS DDAISNILSK SSTQLTLEEL QNPDNRKQIT IDDLKDENKR KELITKIKNK    440
DRB1*12:02                                                                                                             **** **********
DRB1*15:02            ********** *******
DQA1*01:01/DQB1*03:01                        ********* ********** **    **** ********** ****
DQA1*01:01/DQB1*03:03
DQA1*01:02/DQB1*03:01                        ********* ********** **
DQA1*01:02/DQB1*03:03                        ********* ********** **
DQA1*03:02/DQB1*03:01                        ********* ********** ********** ********** ***
DQA1*03:02/DQB1*03:03                        ********* ********** *       ** ********** *****
DQA1*06:01/DQB1*03:01                        ********* ********** **
DQA1*06:01/DQB1*03:03                        ********* ********** **      ** ********** *****
DQA1*06:01/DQB1*05:01 ********** *****                                     * ********** *********
DQA1*06:01/DQB1*05:02
```

```
                      ITEEEGKLNA LKGDVDSKLE KFKKIEGEFK PLLEKFYDER LDNSITTENF EKFLSKRTEY LTEKNLLESS SYELSKALVK KLKKQLMYLE DYSLRKEVFD EEVNHFNCLD   550
DRB1*12:02            *****                                             **** ********** ***     ** ********** ********** ***
DRB1*15:02                                                                                                                           **** **********
DQA1*01:01/DQB1*03:01
DQA1*01:01/DQB1*03:03
DQA1*01:02/DQB1*03:01
DQA1*01:02/DQB1*03:03
DQA1*03:02/DQB1*03:01
DQA1*03:02/DQB1*03:03
DQA1*06:01/DQB1*03:01
DQA1*06:01/DQB1*03:03
DQA1*06:01/DQB1*05:01
DQA1*06:01/DQB1*05:02
```

```
                      LQLNADIHKL ESEIKRKENL LTVVDTLKFS DVVELQVQKV LLGKKIGQLK NVEAFLQKAK LKETFHIPQA YGTGEQSEPY YLIALKREID KLNISIPKIE EMLKNEKKLE   660
DRB1*12:02                                             ********** ********** ********** *******       ******* ********** *****
DRB1*15:02            *******
DQA1*01:01/DQB1*03:01
DQA1*01:01/DQB1*03:03
DQA1*01:02/DQB1*03:01
DQA1*01:02/DQB1*03:03
DQA1*03:02/DQB1*03:01
DQA1*03:02/DQB1*03:03
DQA1*06:01/DQB1*03:01
DQA1*06:01/DQB1*03:03
DQA1*06:01/DQB1*05:01                ****** **********
DQA1*06:01/DQB1*05:02
```

```
                                         →← Block VI
                      EEKIKAAAQN VSGHVSGADE TSNSHGSSGG GGSTQTVTTT PSTTTTATTS SQTVSVGETG SAQAQAQPQP QPQPQPQTQT QTQTQPQAAG ATGTPGQSGQ SGQSGQSGAE   770
DRB1*12:02
DRB1*15:02
DQA1*01:01/DQB1*03:01                                                               ********* **********
DQA1*01:01/DQB1*03:03
DQA1*01:02/DQB1*03:01 ********** *****                            ********** ********** ********** *
DQA1*01:02/DQB1*03:03                                             ********** ********** *********
DQA1*03:02/DQB1*03:01 ********** ********** **                  * ********** ********** ********** **         ********** ********** **********
DQA1*03:02/DQB1*03:03                                           * ********** ********** **********
DQA1*06:01/DQB1*03:01 ********** ********** **         ********** ********** ********** ********** ********** ********** ********** *
DQA1*06:01/DQB1*03:03                                * ********** ********** ********** **********
DQA1*06:01/DQB1*05:01
DQA1*06:01/DQB1*05:02
```

```
                                                                                      →← Block VII
                      GTTETTGTTG QAGTTGTPEQ AAAAGPQAET TATPGQAGAA GAAGPQAETT GTPGQAGAAG PQTETEVEET QEIGIVVPTL SKLQYLEKLY DFLKTAYVCH INILVNNSTM   880
DRB1*12:02                                                                                    ********* ********** ********** *       **
DRB1*15:02                                                                                                                     ********** **********
DQA1*01:01/DQB1*03:01          * ********** ********** ********** ********** **
DQA1*01:01/DQB1*03:03
DQA1*01:02/DQB1*03:01          * ********** ********** ********** ********** ***               *** ********** ***
DQA1*01:02/DQB1*03:03                 ***** ********** ***                                    **** ********** ***
DQA1*03:02/DQB1*03:01 ********** ********** ********** ********** ********** ********** ********** ********** ***
DQA1*03:02/DQB1*03:03                ****** ********** ********** **********                  **** ********** ****
DQA1*06:01/DQB1*03:01 ********** ********** ********** ********** ********** ********** *     **** ********** ****
DQA1*06:01/DQB1*03:03                ****** ********** ****                                   **** ********** ****
DQA1*06:01/DQB1*05:01                                                                       ** ********** ******
DQA1*06:01/DQB1*05:02
```

```
                                                                                                                                                    →←
                      NETLLQQYKL KIEEDKKLLE KCDQLDLLFN VQNNLQVMYS MYDSVSNVLQ NQYKELNQKE MIYNIYKLVK KNDKLKNFLN LTANSAAASS ALPPPPSVPP AVPPASQQPQ   990
DRB1*12:02            ********** ****                                             ***** ********** ********** ********
DRB1*15:02            *                       ******** ********** ********** ***    **** ********** ********** *********
DQA1*01:01/DQB1*03:01                                                                                 ******* ********** **********        ***
DQA1*01:01/DQB1*03:03                                                                                    **** ********** ****
DQA1*01:02/DQB1*03:01                                                                                 ******* ********** *********         ***
DQA1*01:02/DQB1*03:03                                                                                 ******* ********** ********          ***
DQA1*03:02/DQB1*03:01                                                                                 ******* ********** ********** **********
DQA1*03:02/DQB1*03:03                                                                                 ******* ********** ********** **********
DQA1*06:01/DQB1*03:01                                                                                 ******* ********** **********        ***
DQA1*06:01/DQB1*03:03                                                                                 ******* ********** ********** **********
DQA1*06:01/DQB1*05:01                                   ******** ********** ********** *                       ******** ********** **
DQA1*06:01/DQB1*05:02

                      Block VIII                                  →← Block IX
                      PQAALPAQPQ AAVPAQSQAT VPAQSQAAVP ATTQSSSVSA PTGTNGASPA TPVAPAGSEN AIQLKANDNE DDANELDFDI DDIYIKYLEQ VSKYDENFKN FIESKKDIIN   1100
DRB1*12:02
DRB1*15:02
DQA1*01:01/DQB1*03:01 ********** ********** ********** ********** ********** ********
DQA1*01:01/DQB1*03:03
DQA1*01:02/DQB1*03:01 ********** ********** ********** ********** ********** ********** ******
DQA1*01:02/DQB1*03:03 ********** ********** ********** ********** **
DQA1*03:02/DQB1*03:01 ********** ********** ********** ********** ********** ********** ******
DQA1*03:02/DQB1*03:03 ********** ********** ********** ********** ********** ********** ****
DQA1*06:01/DQB1*03:01 ********** ********** ********** ********** ********** ********** *********
DQA1*06:01/DQB1*03:03 ********** ********** ********** ********** ********** ********** *
DQA1*06:01/DQB1*05:01
DQA1*06:01/DQB1*05:02

                      KMSESEWKEL GEEINTLKQD IQSSFDNFGK YKLKLERLLK KKNKITSSTN HIKEYSILKA QLLRKKNILN NPRHVLAAFV VFFNKKIEAE KKEVENALKN TDIMLKYYKA   1210
DRB1*12:02                                     ******* ********** ********** ********** ********** ********** ********** **   ***** **********
DRB1*15:02                                                                 ***** ********** ******        ******** ********** ***               ***
DQA1*01:01/DQB1*03:01
DQA1*01:01/DQB1*03:03
DQA1*01:02/DQB1*03:01
DQA1*01:02/DQB1*03:03
DQA1*03:02/DQB1*03:01
DQA1*03:02/DQB1*03:03                                                                                                                         *
DQA1*06:01/DQB1*03:01
DQA1*06:01/DQB1*03:03
DQA1*06:01/DQB1*05:01                                                                                                                       ***
DQA1*06:01/DQB1*05:02

                      RTKYYISEAF PLKTITEQSL QKEINYLHLE KFKVYSRLEG RIKKMLNLEK ENITYLSGGL HHVLTELKEI INDKTYTGYT HTKNNEEVNK ALNVYEELLP KQISTEEQPD
DRB1*12:02            ********** *****   ** ********** ********** ********** ********** ****
DRB1*15:02            ********** ********        ***** ********** ******
DQA1*01:01/DQB1*03:01 ********** *******
DQA1*01:01/DQB1*03:03
DQA1*01:02/DQB1*03:01
DQA1*01:02/DQB1*03:03
DQA1*03:02/DQB1*03:01
DQA1*03:02/DQB1*03:03 ********** *******
DQA1*06:01/DQB1*03:01
DQA1*06:01/DQB1*03:03
DQA1*06:01/DQB1*05:01 ********** *******
DQA1*06:01/DQB1*05:02
```

```
                       NALADGTENA TEGAEVRAAT AESLVQGEDE YPEEVDEVIV FPIVGKKEKE NPLDQITKGQ AETKQDDNIL KPITNEYEVL YIKPLAGVYR VLRKQIGDQI DAFNSNLTNA   1430
DRB1*12:02                                                                                             ******* ********** ********** **
DRB1*15:02
DQA1*01:01/DQB1*03:01      ****** ********** *********
DQA1*01:01/DQB1*03:03
DQA1*01:02/DQB1*03:01                 ****** **********
DQA1*01:02/DQB1*03:03       ***** ********** **********                                                                            * **********
DQA1*03:02/DQB1*03:01      ****** ********** **********
DQA1*03:02/DQB1*03:03      ****** ********** **********
DQA1*06:01/DQB1*03:01      ****** ********** **********
DQA1*06:01/DQB1*03:03       ***** ********** **********
DQA1*06:01/DQB1*05:01                                                                                  ******* ********** **
DQA1*06:01/DQB1*05:02

                       LDTRKKKRTY FLDVLNSDLI QFKHATSDSY IIKDPYKLLD VDKKAKLIGS YKYIVSAIEK DITSAENGVE YYDKMTKLYK TQLEAVKSAI AEAQKEGDKK TENEKYIPFL   1540
DRB1*12:02                                                         ********** **********         ** ********** ******
DRB1*15:02                                                          ********* ********** **
DQA1*01:01/DQB1*03:01                                                                                         ** ********** *********
DQA1*01:01/DQB1*03:03
DQA1*01:02/DQB1*03:01                                                                                         ** ********** *********
DQA1*01:02/DQB1*03:03  ****                                                                                   ** ********** *********
DQA1*03:02/DQB1*03:01                                                                                         ** ********** *********
DQA1*03:02/DQB1*03:03                                                                                        *** ********** *********
DQA1*06:01/DQB1*03:01                                                                                         ** ********** *********
DQA1*06:01/DQB1*03:03                                                                                        *** ********** ********** **********
DQA1*06:01/DQB1*05:01       ***** ********** ********** ********** ********** ********** **                                            ********
DQA1*06:01/DQB1*05:02

                       TNMQTLYENL LNKINGNIIN LKTLITNCNL EKDAVNITIS KLTEYSKFDE KIEMFKNSKN EKDIASSGIL DILKQKGLVN KNESTKIISE LLGVDSNALL NISAKHACTE   1650
DRB1*12:02                          ******** ********** ********** ********** ********** ********** ********** ***
DRB1*15:02
DQA1*01:01/DQB1*03:01
DQA1*01:01/DQB1*03:03
DQA1*01:02/DQB1*03:01
DQA1*01:02/DQB1*03:03                                                                 ** ********** *****
DQA1*03:02/DQB1*03:01
DQA1*03:02/DQB1*03:03
DQA1*06:01/DQB1*03:01
DQA1*06:01/DQB1*03:03  ********** ********** ********** ********** ********** ********** ********** *****
DQA1*06:01/DQB1*05:01  ********** **                                                                                ***** ********** ****
DQA1*06:01/DQB1*05:02

                       TKYPENAGCY RYEDGKEVWR CLLNYKLVDG GCVEDEEPSC QVNNGGCAPE ANCTKGDDNK IVCACNAPYS EPIFEGVFCG SSSFLGLSLL LAALLIMFNL L          1751
DRB1*12:02                          ******** ********** *
DRB1*15:02                          ******** ********** ***                                                *** ********** ********
DQA1*01:01/DQB1*03:01
DQA1*01:01/DQB1*03:03
DQA1*01:02/DQB1*03:01
DQA1*01:02/DQB1*03:03                                                                                         ** ********** *****
DQA1*03:02/DQB1*03:01
DQA1*03:02/DQB1*03:03
DQA1*06:01/DQB1*03:01
DQA1*06:01/DQB1*03:03
DQA1*06:01/DQB1*05:01
DQA1*06:01/DQB1*05:02
```

Supplemental Figure S3. Schematic representation of malarial MSP1s, depicting homologous regions (broken lines) across *Plasmodium s*pecies. Open and filled boxes are conserved sequences and variable non-repeat regions within each locus. Boxes with checkerboard, horizontal brick, solid diamond and horizontal line are repeat regions. The schemes are drawn in approximate proportion to the gene length.

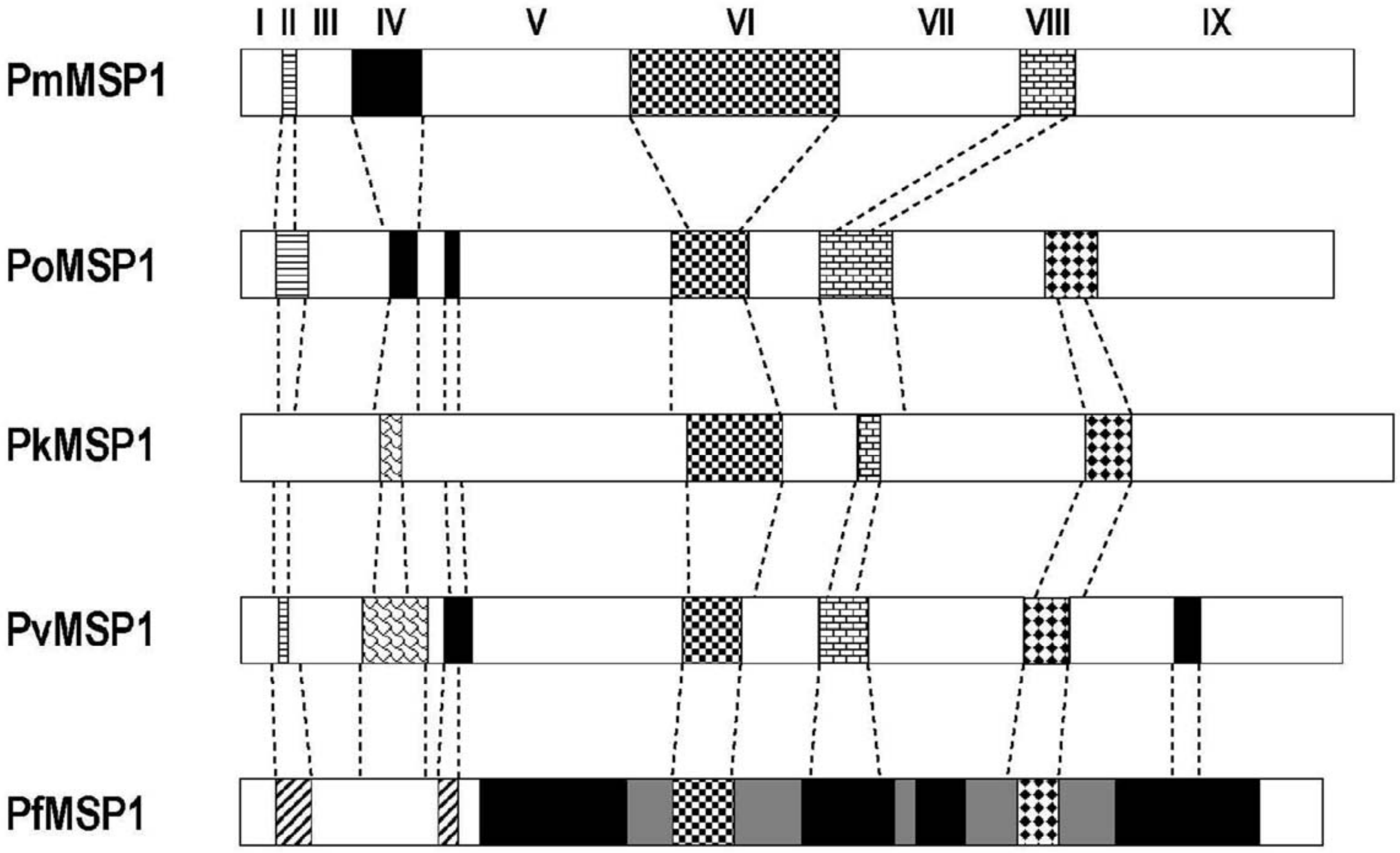

Supplemental Figure S4. Quasi-palindromic sequences around the insertion/deletion region at the 3’ portion of *PmMSP1* and *PvMSP1*.

PmMSP1

```
                111111111111111111111111111111111111111111111111111111111111111111111111122222222222222222222222222222222222222222222222
                222333333333344444444445555555555666666666677777777778888888888999999999900000000001111111111222222222233333333334444444
                789012345678901234567890123456789012345678901234567890123456789012345678901234567890123456789012345678901234567890123456
Thai_PM1        TATAACCTATTTCACAAAAAAAAATTTGCCTTAACAGACATTAATAAGGATGGTAATACCAGTACTACTAATGCAAATAATGCAAATAATAAAGATGATAGTAAGGTTTCCTCCGTAACA
Thai_PM2        ..................................................................---------.C...........................................
Thai_PM35       ........................................................................................................................
Thai_PM20       ..................................................................---------.C.G.........................................
Thai_PM9        .....................................................................------.C...........................................
Thai_PM13       ..........................................---------------------------------.C...........................................
KC906713        ...........................................C.------------------------------...GCAAAT....................................
KC906712        ...........................................C.------------------------------G..GCAAAT....................................
AF138880        .......................................................G..........---------.C.G................................C........

                222222222222222222222222222222222222222222222222222223333333333333333333333333333333333
                444555555555566666666667777777777888888888899999999990000000000111111111122222222223333
                789012345678901234567890123456789012345678901234567890123456789012345678901234567890123
Thai_PM1        GCAAAAATAGGAAATTTTGTTAGCAAAGTTCTAAACCTGAATTTGCCAGGTTATGTTCAGTTAACTTTTTCAATTAGGGAATTAATT
Thai_PM2        .......................................................................................
Thai_PM35       .......................................................................................
Thai_PM20       .......................................................................................
Thai_PM9        .......................................................................................
Thai_PM13       .......................................................................................
KC906713        .......................................................................................
KC906712        .......................................................................................
AF138880        .......................................................................................
```

PvMSP1

```
                111111111111111111111111111111111111111111111111111111111111111111111111111111122222222222222222222222222222222222222222
                222222222333333333344444444445555555555666666666677777777778888888888999999999900000000001111111111222222222233333333334
                123456789012345678901234567890123456789012345678901234567890123456789012345678901234567890123456789012345678901234567890
TG55            GGCTACGAGCTCTTCCACAAAAAAAAGTTAGGAGAAAATGATATTAAGGTAGATGCTAAT------GCAAATAATAATAATAACAATCAGGTTAGCGTTTTAACTTCCAAAATAAGAAAT
T077            .....................................................A......------......................................................
T064            .....................................................AA.C...GCTAGT....................................G.................
```

Note: Positions are after the alignment. Quasi-palindromic sequences are highlighted in light blue and green for *PmMSP1* and yellow for *PvMSP1*. Identical sequences and deletions are shown as dots and dashes. Distinct alleles spanning the indel region are shown.
